# Delivery Methods for RNAi in Mosquito Larvae

**DOI:** 10.1093/jisesa/ieaa074

**Published:** 2020-07-29

**Authors:** Kashif Munawar, Azzam M Alahmed, Sayed M S Khalil

**Affiliations:** 1 Plant Protection Department, College of Food and Agricultural Sciences, King Saud University, Riyadh, Saudi Arabia; 2 Agricultural Genetic Engineering Research Institute, Agricultural Research Center, Giza, Egypt

**Keywords:** mosquito control, RNAi, delivery, yeast, nanoparticles

## Abstract

Mosquito-transmitted diseases pose a threat for a great portion of the world population. Chemical insecticides are the main tool for mosquito control. Heavy dependence on chemicals created several problems such as resistance development in many mosquito species, environmental effects, and human health issues. Other tools for mosquito control were developed and used in some parts of the world. Ribonucleic acid interference (RNAi) is a reverse genetic mechanism that was recently introduced as a new tool for pest control. Regarding mosquito, RNAi was used to study gene function and to discover genes that can be used as targets for control purposes. Several delivery methods are used to induce RNAi in mosquito larvae. Some methods such as injection and soaking are used routinely in RNAi research but have no application in the field. Other methods such as nanoparticles and microbes have some characteristics that make them good candidates for field application. In this report, we will focus on delivery methods for RNAi in mosquito larvae and will give examples for each method.

The family Culicidae contains 3,570 valid species of mosquitoes including disease vectors such as *Anopheles*, *Aedes*, and *Culex* (Harbach 2020). These mosquitoes are the vectors of numerous deadly diseases such as malaria, Zika fever, dengue fever, yellow fever, chikungunya, West Nile fever, and filariasis. About half of the world population is at risk of dengue with an estimated 100–400 million infections and 22,000 deaths each year (CDC 2020). In 2018, malaria alone caused 405,000 deaths mostly in children (WHO 2020). With every passing day, mosquitoes and mosquito-borne diseases are becoming more and more challenging because of their ability to adapt and thrive in every changing condition.

Mosquito control is a key component of disease control especially in the absence of effective vaccine or a cure for some mosquito-borne diseases (Bourtzis et al. 2016). Chemical insecticides are the major means for mosquito control but their excessive use caused mosquito to develop resistance to almost all known classes of insecticides (Liu 2015, Rubert et al. 2016). As mosquito resistance toward chemicals is becoming more challenging, alternative management tactics have been developed such as environmental modification, biological control, new methods of using chemical insecticides, and genetic modification of mosquito (summarized in McGraw and O’Neill 2013, Bourtzis et al. 2016).

Recently, reverse genetic studies based on ribonucleic acid interference (RNAi) have become an important tool to study gene function in living organisms through knockdown of the target genes (Fire et al. 1998). The process starts with the introduction of double-stranded RNA (dsRNA) into the living cell which cleaves it by a specific RNase (Dicer) into short interfering RNAs (siRNAs) of size ~21 nt. These siRNAs are then added into ~550 kDa RNA-induced silencing complex (RISC), which degenerate the complementary mRNA, thus preventing the translation process (Fjose et al. 2001, Agrawal et al. 2003).

Insects have the basic components that run the RNAi mechanism and there are many successful examples of RNAi in different insect orders (Zhu and Palli 2020). The ability to downregulate target genes in insects led to proposing RNAi as a new tool for insect control (Baum et al. 2007, Mao et al. 2007). Regarding mosquito, RNAi has been used extensively in functional characterization of mosquito genes and in studying the mosquito–pathogen interaction (summarized in Airs and Bartholomay 2017). RNAi in mosquito focused on the adult stage and the main delivery method was the direct injection into the body cavity. RNAi in adult mosquito through injection is a well-established method used routinely in mosquito research. Many reports showed the success of utilizing RNAi in the silencing of genes related to different physiological processes and in many tissues and organs in different mosquito species (Drake et al. 2015, Lamacchia et al. 2013, Kim et al. 2020, Wu et al. 2020, and many others). Publication of the full genome sequence of different species such as *An. gambiae* (Holt et al. 2002), *Ae. aegypti* (Nene et al. 2007), and *Cx. quinquefasiatus* (Arensburger et al. 2010) enabled researchers to identify more genes and to study their function through molecular tools such as RNAi. Recently, RNAi in mosquito larvae was achieved and showed success in downregulation of a lot of target genes. This downregulation was confirmed through molecular techniques such as qRT-PCR and phenotypic changes such as mortality. As in other insects, delivery method is a key player in the success of RNAi in mosquito larvae (Whitten 2019). Several methods such as injection, soaking, nanoparticles, and microbial-based were adapted to deliver dsRNA into mosquito larvae. To the best of our knowledge, there are two reviews that discussed different aspects of RNAi in mosquito (Airs and Bartholomay 2017, Balakrishna Pillai et al. 2017). Recently, Whitten (2019) discussed different methods for RNAi delivery in medical and veterinary pests. In spite of the importance of RNAi in mosquito larvae and increasing the number of published studies in recent years, there is no comprehensive collection of publications regarding this topic. Based on that, we will focus on the delivery methods for RNAi in mosquito larvae and will give examples of each method. A comprehensive collection of publications showing delivery methods and target genes will be presented in Table 1. In general, we divided delivery methods into two main categories; nonmicrobial and microbial (presented in Fig. 1). Nonmicrobial delivery of the RNAi trigger (dsRNA, miRNA, shRNA) takes place through soaking, nanoparticles, injection, or osmotic treatment (dehydration and rehydration). On the other hand, microbial methods utilize microbes (bacteria, yeast, algae, and viruses) for both synthesis and delivery of the RNAi trigger. RNAi in mosquito larvae showed both local and systemic effects, for example, introduction of dsRNA through the larval gut affected genes in the gut cells as well as in other tissues such as the nervous system. It is worth mentioning that RNAi in larvae showed downregulation of target genes in both larval and adult stages. Such results give an opportunity to incorporate RNAi in some control programs targeting not only the larval stage but also the adult stage such as sterile insect technique (SIT).

**Table 1.** Delivery methods, mosquito species, target genes, and RNAi trigger molecules

Delivery method	Species	Target gene	RNAi trigger	Reference
Soaking	*Ae. aegypti*	*β-tubulin, chitin synthase-1 and -2 (CHS1, 2), heat shock protein 83 (hsp83)*	dsRNA	Singh et al. (2013)
	*Ae. aegypti*	*P-glycoprotein*	dsRNA	Figueira-Mansur et al. (2013)
	*Ae. aegypti*	*Methionine selective transporter (NAT5)*	dsRNA	Meleshkevitch et al. (2013)
	*Ae. aegypti*	*growth arrest-specific protein 8 (gas8), boule (bol), fuzzy onions (fzo), no-hitter (nht), zero population growth (zpg), female-specific doublesex (dsxF)*	dsRNA	Whyard et al. (2015)
	*Ae. aegypti*	*Voltage-gated sodium channel (VGSC), cytochrome P450, P-glycoprotein*	dsRNA	Bona et al. (2016)
	*Ae. aegypti*	*fasciculation and elongation protein zeta 2 (fez2), leukocyte receptor cluster (lrc) member*	siRNA	Hapairai et al. (2017)
	*An. gambiae*	*suppressor of actin (sac1), offtrack (otk), lrc*	siRNA	Mysore et al. (2017)
	*Ae. aegypti*, *Ae. albopictus*, *An. gambiae*, *Cx. quinquefasciatus*	*synaptotagmin (syt)*	siRNA	Mysore et al. (2019a)
	*Ae. aegypti*, *Ae. albopictus*, *An. gambiae*, *Cx. quinquefasciatus*	*semaphorin-1a (sema1a)*	siRNA	Mysore et al. (2019b)
	*Ae. aegypti*	*CHSA, B*	dsRNA	Lopez et al. (2019)
	*An. stephensi*	*ATP binding cassette transporters 4 (ABCG4)*	siRNA	Negri et al. (2019)
	*Ae. aegypti*	*dopamine 1 receptor (dop1)*	siRNA	Hapairai et al. (2020)
Nanoparticles	*An. gambiae*	*CHS1, 2*	dsRNA	Zhang et al. (2010)
	*Ae. aegypti*	*Mitogen-activated protein kinase p38 (MAPK p38)*	dsRNA	Cancino-Rodezno et al. (2010)
	*Ae. aegypti*	*ATP synthase beta subunit, actin and hsp90*	dsRNA	Cancino-Rodezno et al. (2012)
	*Ae. aegypti*	*GPI-anchored alkaline phosphatase (alp1)*	dsRNA	Jimenez et al. (2012)
	*Ae. aegypti*	*Cadherin (cad)*	dsRNA	Rodríguez-Almazán et al. (2012)
	*Ae. aegypti*	*inositol requiring enzyme 1(ire-1), x-box binding protein (xbp-1), caspase-1, scap, s2p*	dsRNA	Bedoya-Pérez et al. (2013)
	*Ae. aegypti*	*sema1a*	siRNA	Mysore et al. (2013), (2014b)
	*Ae. aegypti*	*single-minded (sim)*	siRNA	Mysore et al. (2014a)
	*Ae. aegypti*	*vacuolar-sorting protein (snf7), steroid receptor coactivator (src)*	dsRNA	Das et al. (2015)
	*An. gambiae*	*Cad1, Cad2*	siRNA	Zhang et al. (2015a)
	*Ae. aegypti*	*dsx*	siRNA	Mysore et al. (2015)
	*Ae. aegypti*	*wing development vestigial (vg)*	dsRNA	Ramesh Kumar et al. (2016)
	*Ae. aegypti*	*fez2 and lrc*	siRNA	Hapairai et al. (2017)
	*Ae. aegypti*	*inhibitor of apoptosis (iap), snakeskin (ssk), helicase at 25e (hel25e), membrane-spanning protein (mesh), snf7, src, lrc, otk, sac1*	dsRNA	Dhandapani et al. (2019)
	*Ae. aegypti*	*3,4-dihydroxyphenylacetaldehyde (DOPAL) synthase*	dsRNA	Chen et al. (2019)
Dehydration and rehydration	*Cx. pipiens*	*hsp90*	dsRNA	Lopez-Martinez et al. (2012)
Injection	*Ae. aegypti*	*sterol carrier protein-2 (scp-2)*	dsRNA	Blitzer et al. (2005)
	*An. gambiae*	*odorant receptor7 (or7), or40, ionotropic receptor (ir76b)*	siRNA	Liu et al. (2010)
	*An. gambiae*	*Transient receptor potential subfamily A1 (TRPA1) channel*	siRNA	Liu and Zwiebel (2013)
	*An. gambiae*	*sac1, lrc, otk*	siRNA	Mysore et al. (2017)
	*Ae. aegypti*	*fez2 and lrc*	siRNA	Hapairai et al. (2017)
Bacterial delivery	*Ae. aegypti*	*growth arrest-specific protein 8 (gas8), boule (bol), fuzzy onions (fzo), no-hitter (nht), zero population growth (zpg), female-specific doublesex (dsxF)*	dsRNA	Whyard et al. (2015)
	*Ae. aegypti*	*wing development vestigial (vg)*	dsRNA	Ramesh Kumar et al. (2016)
	*An. gambiae*	*sac1, lrc, otk*	siRNA	Mysore et al. (2017)
	*Ae. aegypti*	*fez2 and lrc*	dsRNA	Hapairai et al. (2017)
	*An. gambiae*	*dsxF*	dsRNA	Taracena et al. (2019)
	*Ae. aegypti*	*CHSA, B*	dsRNA	Lopez et al. (2019)
Algal delivery	*An. stephensi*	*3-hydroxy kynurenine transaminase (3-HKT)*	dsRNA	Kumar et al. (2013)
Viral delivery	*Ae. albopictus*	*v-ATPase* subunit A	shRNA	Gu et al. (2011)
Yeast delivery	*Ae. aegypti*	*JH acid methyltransferase (jhamt)*	lhRNA	Van Ekert et al. (2014)
	*Ae. aegypti*	*fez2 and lrc*	shRNA	Hapairai et al. (2017)
	*An. gambiae*	*sac1, lrc, otk*	siRNA	Mysore et al. (2017)
	*Ae. aegypti*, *Ae. albopictus*, *An. gambiae*, *Cx. quinquefasciatus*	*syt*	shRNA	Mysore et al. (2019a)
	*Ae. aegypti*, *Ae. albopictus*, *An. gambiae*, *Cx. quinquefasciatus*	*sema1a*	shRNA	Mysore et al. (2019b)
	*Ae. aegypti*, *Ae. albopictus, An. gambiae*	*dopamine 1 receptor (dop1)*	shRNA	Hapairai et al. (2020)

**Fig. 1.**
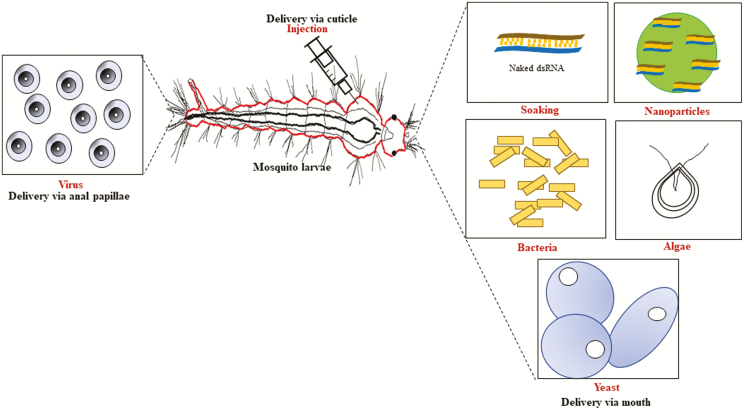
Different delivery methods used for RNAi in mosquito larvae.

## Nonmicrobial Delivery Methods

Nonmicrobial delivery includes production of RNAi trigger (usually in a cell-free system such as commercial kits or by chemical synthesis) and delivery through one of the methods mentioned below. Some methods are easy and do not need extra chemicals or equipment such as soaking. Others are laborious, time-consuming, and cause injury to the treated insects such as injection. Nanoparticles should be prepared first, then complexed with the dsRNA and incorporated with food to attract larvae.

### Soaking

Larval soaking is the most simple and straightforward delivery method as it does not need specific equipment and has no injurious effect on insects. Larvae are soaked in a small volume of the naked RNAi trigger for a specific time, then transferred to the normal rearing conditions. RNAi through soaking of mosquito larvae was first performed by Singh et al. (2013). The study showed the successful knockdown of β-tubulin, chitin synthase-1 and -2, and heat shock protein 83 genes by soaking first-instar *Ae. aegypti* larvae in dsRNA solution for only 2 h. They used the same soaking technique for *Drosophila melanogaster* larvae using β-tubulin dsRNA either from *Ae. aegypti* or from *D. melanogaster* and concluded that the significant mortality was observed in the larvae that were treated with their own gene, confirming that the RNAi effect is species-specific. They proved that dsRNA in the soaking solution passed through the gut by adding a dye to the solution and photographing the treated larvae using a color video camera. However, this does not exclude the possibility of cuticle transport (Whyard et al. 2015). Meleshkevitch et al. (2013) soaked eggs in the dsRNA solution allowing neonates to hatch directly into the solution. Figueira-Mansur et al. (2013) treated second-instar larvae by soaking in the dsRNA solution for 24 h. Whyard et al. (2015) showed that larval treatment can be used in targeting adult-specific genes. They soaked *Ae. aegypti* larvae in spermatogenesis-specific dsRNA solution for 1 h/day for 6 d, a treatment that achieved high levels of male sterility. They also targeted the female-specific doublesex gene and produced a population of 97% adult males. Other genes were successfully downregulated through soaking such as voltage-gate sodium channel and cytochrome P450 (Bona et al. 2016), chitin synthase A and B (Lopez et al. 2019), and others. Soaking was also adapted for gene downregulation in other mosquito species such as *An. gambiae* (Mysore et al. 2017) and *An. stephensi* (Negri et al. 2019). Larval soaking is a simple and easy technique but not applicable in the field as it needs the application of huge amounts of dsRNA to ensure delivery. Also, a study by Fischer et al. (2017) showed a short half-life of naked dsRNA in aquatic environment.

### Injection

Injection is the most common way to introduce dsRNA into insect bodies. Injection ensures delivery into the hemocoel, distribution of dsRNA through body organs and tissues, and bypassing some body barriers such as cuticle and gut. Regarding mosquitoes, dsRNA delivery through injection is common in pupal and adult stages (Regna et al. 2016, Isoe et al. 2019); however, it was used in few cases in larval stages. In general, the injection process includes anaesthetizing larvae in cold environment followed by using lab-made capillary glass needle for injecting few nanoliters of the dsRNA solution into the thoracic region. Blitzer et al. (2005) injected the sterol carrier protein-2 dsRNA into fourth instars of *Ae. aegypti* and studied the knockdown effect in pupae and adults. Successful knockdown of the target gene resulted in high adult mortality and low egg viability. Liu et al. (2010) and Liu and Zwiebel (2013) used larval injection to study the olfactory signaling pathways and peripheral thermosensory response in *An. gambiae*, respectively. Although direct injection of dsRNA into the body cavity has a great benefit in RNAi research but clearly has no field application.

### Nanoparticles

Nanoparticles have been widely used in medicine for drug delivery as well as siRNA therapy (Chenthamara et al. 2019). Some nanoparticles are biodegradable, cost-effective, provide sustained release, and have no side or environmental effects. Chitosan is economical and biodegradable material used in drug delivery for a long time (Mohammed et al. 2017). Recently, chitosan has been used in dsRNA delivery to insects as it provides protection from digestion by nucleases and stability in high pH of insect gut. Chitosan also has antimicrobial activity that may protect dsRNA from degradation by microbes (Jeon et al. 2014).

Zhang et al. (2010) were the first to use chitosan nanoparticles/dsRNA (CNP/dsRNA) in feeding mosquito larvae to induce RNAi. The attraction forces between positive charges on chitosan amino acids and the negative ones carried by the phosphate groups of dsRNA allow the self-assembly of the CNP/dsRNA complex. Synthesis of the CNP/dsRNA is explained in details in Zhang et al. (2010) and Zhang et al. (2015a,b). After formation of the CNP/dsRNA, the complex is entrapped with mosquito food in agarose gel and introduced to the larvae every day until consumed. Using CNP/dsRNA, Zhang et al. (2010) could downregulate two chitin synthase genes in the malaria mosquito *An. gambiae*. Later, RNAi through nanoparticles was widely used in mosquito research to study the function of different genes such as cadherin (Rodríguez-Almazán et al. 2012, Zhang et al. 2015a), wing-development vestigial genes (Ramesh Kumar et al. 2016), and many others.

Other nanoparticles such as carbon quantum dots (CQDs) and silica nanoparticles (SNP) were also tested as dsRNA delivery vehicles. In one comparative study, Das et al. (2015) used the three nanoparticles CNP, CQDs, and SNP to induce RNAi of two target genes, *SNF7* and *SRC*, in *Ae. aegypti*. They found that CQDs gave more gene silencing and larval mortality compared to the other two nanoparticles. This may be due to the stability of the CQD/dsRNA complex in extreme pH of the mosquito gut and the rapid distribution through the insect body. To improve dsRNA binding and delivery of CNP, Dhandapani et al. (2019) cross-linked chitosan to sodium tripolyphosphate, a treatment that increased the overall efficacy of CNP in binding, protection, stability, delivery of dsRNA, gene knockdown, and larval mortality.

Liposome is a lipid form of nanoparticles used widely in the biomedical field for siRNA delivery (Li et al. 2020). Cancino-Rodezno et al. (2010) were the first to use liposome to deliver dsRNA to mosquito where they downregulated the MAPK p38 gene in *Ae. aegypti* larvae. Subsequent publications from the same research group showed downregulation of many genes in the same species (Cancino-Rodezno et al. 2010, Cancino-Rodezno et al. 2012, Jiménez et al. 2012, Rodríguez-Almazán et al. 2012). These researchers found that liposome had some larval toxicity depending on the exposure time and liposome concentration. Yet, there is potential for the use of liposomes-based dsRNA delivery system in insects as it may limit dsRNA degradation and increase the delivery through gut cells (Singh et al. 2013).

### Dehydration and Rehydration

Salinity tolerance was utilized by Lopez-Martinez et al. (2012) to introduce dsRNA into *Cx. pipiens* larvae. They dehydrated the fourth-instar larvae in 2 M NaCl solution for 30 min followed by rehydration in dsRNA solution for 2 h. By this method, they were able to reduce the transcription of *hsp90* not only in larvae but also in pupal and adult stages. While this method is suitable for salinity-tolerant larvae such as the *Culex* fourth instars, it is not suitable for other species and even smaller *Culex* larvae because they cannot recover from such harsh osmotic insult.

## Microbial Delivery Methods

A major advantage of microbes is using them for both synthesis and delivery of RNAi-triggering molecules. Molecular tools facilitated the genetic modification of microbes and mass culturing facilitated their production on large scale. Some microbes such as yeast do not possess the RNAi machinery which allows the accumulation of large amounts of RNAi-triggering molecules (Duman-Scheel 2019).

### Bacterial Delivery

Timmons and Fire (1998) were the pioneers who used the bacterial-based dsRNA synthesis and delivery to the nematode *Caenorhabditis elegans*. To produce dsRNA, they inserted the target gene sequence between two opposite copies of the T7 promoter carried on the L4440 plasmid and transformed the RNase III-deficient *E. coli* strain HT115 (DE3). Later, the technique was used to induce RNAi in insects from different orders such as the beet armyworm, *Spodoptera exigua* (Tian et al. 2009), Colorado potato beetle, *Leptinotarsa decemlineata* (Zhu et al. 2011), and the mosquito *Ae. aegypti* (Lopez et al. 2019). Whyard et al. (2015) embedded alive or heat-killed bacteria with mosquito food into agar cubes and used it to feed *Ae. aegypti* larvae till pupation. They did not notice significant loss in the RNAi efficacy of the killed bacteria. Lopez et al. (2019) downregulated two chitin synthase genes and induced mortality of *Ae. aegypti* larvae using the same bacterial system but with one modification, i.e., they lysed the bacterial cells and added the lysate to the rearing water. Taracena et al. (2019) adapted the same method to knockdown the female doublesex gene in *An. gambiae*. Treatment of larvae with the modified bacteria affected the adult stage as it reduced the number of adult females and also affected their fecundity. It is worth mentioning that gene knockdown was sex-specific where treatment had no effect on adult males.

### Yeast Delivery

Genetically engineered yeast can be used to produce and deliver short or long hairpin RNA to mosquito larvae. In one study, Van Ekert et al. (2014) transformed the *Pichia pastoris* yeast cells by modified pPicZB plasmid containing long hairpin sequence of *Ae. aegypti* juvenile hormone acid methyltransferase (JHAMT) gene. Feeding larvae on the modified yeast cells resulted in up to 90% knockdown of the target gene as well as mortality in larval, pupal, and adult stages. This leading study was followed by more studies using the well-characterized baker’s yeast *Saccharomyces cerevisiae*. Hapairai et al. (2017) used *S. cerevisiae* to produce shRNA of two target genes (*fez2* and *lrc*) of *Ae. aegypti*. Larvae fed on engineered yeast cells had disrupted neural development and high mortality. Mysore et al. (2017) adapted the same technique for the production and delivery of siRNAs against three genes (*Sac1*, *lrc*, and *otk*) to *An. gambaei* larvae. Using live, killed, and dried killed yeast cells successfully downregulated the target genes and caused high mortalities in mosquito larvae. Later, the same research group modified yeast cells to downregulate several genes in different mosquito species (Mysore et al. 2019a, b; Hapairai et al. 2020).

### Algal Delivery

Kumar et al. (2013) used the unicellular microalga *Chlamydomonas reinhardtii* to produce and deliver dsRNA to mosquito larvae. They stably transformed the algal chloroplast to produce a 328-bp dsRNA of the *An. gambiae* 3-hydroxykynurenine transaminase (3-HKT) gene. *Anopheles gambiae* larvae fed on the transgenic microalga showed reduced levels of 3-HKT gene expression and higher mortality compared to those fed on nontransgenic algal cells. These researchers believe that the potency of the mosquitocide algae can be improved by using microalgae preferred as food for mosquito larvae in their natural habitat.

### Viral Delivery

Gu et al. (2011) successfully used the *Ae. aegypti* densovirus (AeDNV) for the delivery of dsRNA into mosquito cells in vitro and in vivo. They modified the AeDNV genome to produce dsRNA for the V-ATPase gene of the mosquito *Ae. albopictus*. Transfection of C6/C36 cells inhibited up to 90% of the V-ATPase gene expression. *Aedes albopictus* larvae were infected through adding the genetically modified virus to the rearing water for 24 h followed by moving larvae to regular rearing conditions. These researchers believe that the virus entered the larval body through the anal papillae then disseminated to other body organs and tissues. Larvae infected with the modified virus showed nearly 70% reduction in V-ATPase gene expression and higher mortality compared to control larvae. Kolliopoulou et al. (2017) believe that some virus characteristics such as efficiency and specificity make them excellent delivery vehicle. On the other hand, Whitten (2019) mentioned that mutation rates and potential host range should be assessed before using genetically modified viruses in pest control.

### Conclusion

RNAi is a promising tool to incorporate in mosquito control programs. Treatment of larvae can knock down genes in both larval and adult stages. Several genes were proven effective to cause larval and adult mortality. Larval RNAi can be utilized in adult-targeted control programs such as SIT. Some delivery methods such as injection and soaking are excellent research tools while others such as nanoparticles and microbes have potential field applications. However, one of the challenges is the optimization of an economic system able to deliver amounts of dsRNA into larval bodies enough to produce the target gene knockdown level. Gene knockdown depends on many factors such as delivery method, target gene, nature of the RNAi trigger, tissue nature, physiological stage, and cellular mechanisms associated with the RNAi process including enzymatic degradation, cellular uptake and processing, intercellular transport, and intracellular movement. Research on lepidopteran and coleopteran insects showed some differences that partially explained why lepidopterans are more resistant to RNAi. Lepidopterans have higher level of dsRNases, lower intercellular transport, and tendency to trap dsRNA within the endosomal bodies (Shukla et al. 2016, Singh et al. 2017); these factors were not studied in mosquitoes. Only recently, Giesbrecht et al (2020) identified several dsRNases from *Ae. aegypti* and reported that knocking down of gut-specific dsRNases greatly enhanced RNAi in the larvae, a step toward understanding and application of RNAi in mosquito control. It is believed that nanoparticles and microbes are the potential tools for field application (Whitten 2019, Yan et al. 2020). Regarding nanoparticles, there is a need for a delivery system that protects RNA molecules from environmental conditions, does not release them in the aqueous environment, and formulated in such a way to attract mosquito larvae. Microbes are easy to genetically manipulate and have the advantage of both production and protection of RNAi molecules; however, more research is needed for optimization of RNAi molecules expression, production of microbes on economic commercial scale, and formulation that stabilizes them in the environment. Also, shifting to small RNA molecules such as shRNA and siRNA is needed to minimize the nontarget effect. In general, RNAi research in larval mosquito still has a long way to go as number of publications is low, most of the work is not replicated by other labs, and there is a minimal collaboration between different research groups compared to RNAi research on agricultural pests (Mezzetti et al. 2020).
